# HAO1-mediated oxalate metabolism promotes lung pre-metastatic niche formation by inducing neutrophil extracellular traps

**DOI:** 10.1038/s41388-022-02248-3

**Published:** 2022-06-23

**Authors:** Zhicheng Zeng, Shaowan Xu, Feifei Wang, Xin Peng, Wanning Zhang, Yizhi Zhan, Yanqing Ding, Ziguang Liu, Li Liang

**Affiliations:** 1 Department of Pathology, Shunde Hospital, Southern Medical University (The First People’s Hospital of Shunde Foshan), foshan, Guangdong PR China; 2 Department of Pathology, Nanfang Hospital and Basic Medical College, Southern Medical University, Guangzhou, 510515 PR China; 3 Guangdong Province Key Laboratory of Molecular Tumor Pathology, Guangzhou, 510515 Guangdong PR China

**Keywords:** Cancer microenvironment, Metastasis

## Abstract

Metabolic reprogramming has been shown to be involved in cancer-induced pre-metastatic niche (PMN) formation, but the underlying mechanisms have been insufficiently explored. Here, we showed that hydroxyacid oxidase 1 (HAO1), a rate-limiting enzyme of oxalate synthesis, was upregulated in the alveolar epithelial cells of mice bearing metastatic breast cancer cells at the pre-metastatic stage, leading to oxalate accumulation in lung tissue. Lung oxalate accumulation induced neutrophil extracellular trap (NET) formation by activating NADPH oxidase, which facilitated the formation of pre-metastatic niche. In addition, lung oxalate accumulation promoted the proliferation of metastatic cancer cells by activating the MAPK signaling pathway. Pharmacologic inhibition of HAO1 could effectively suppress the lung oxalate accumulation induced by primary cancer, consequently dampening lung metastasis of breast cancer. Breast cancer cells induced HAO1 expression and oxalate accumulation in alveolar epithelial cells by activating TLR3-IRF3 signaling. Collectively, these findings underscore the role of HAO1-mediated oxalate metabolism in cancer-induced lung PMN formation and metastasis. HAO1 could be an appealing therapeutic target for preventing lung metastasis of cancer.

## Introduction

Alteration of microenvironmental signals occurring before the arrival of disseminating cancer cells contributes to distant metastasis of cancer, which is termed the PMN. Metabolic reprogramming is an important characteristic of the PMN that empowers the niche to favor cancer cell colonization and promote metastasis [1]. Primary tumors may modify the metabolic reprogramming of non-tumor cells within the PMN. For example, breast cancer secreted miR-122 reprograms glucose metabolism in the PMN to promote metastasis [2]. However, little is known about how metabolic reprogramming modulates PMN formation.

Oxalate is an inert metabolic end product that is synthesized by a variety of cells, such as liver cells, epithelial cells and apocrine cells [3]. Oxalate accumulation leads to ROS-induced oxidative stress and tissue injury by activating nicotinamide adenine dinucleotide phosphate (NADPH) oxidase [4, 5]. In addition, a previous study has suggested that oxalate promotes the proliferation of breast cancer [6]. HAO1 is a rate-limiting enzyme of oxalate synthesis that catalyzes the oxidation of glycolate to glyoxylate and glyoxylate to oxalate [7, 8]. Pharmacologic inhibition or knockdown of HAO1 is an efficient approach to reduce oxalate production [9–12]. However, the functional role of HAO1-mediated oxalate metabolism in cancer development remains unclear.

Neutrophil extracellular traps (NETs), scaffolds of decondensed chromatin coated with cytotoxic enzymes and proteases, are produced in neutrophils and can be released into the extracellular matrix to trap microorganisms [13]. Pathogen-induced NET formation is primed by activation of the NADPH oxidase enzyme complex [13]. Interestingly, a growing body of evidence suggests that tumor cells are able to stimulate NET formation in the absence of infection, which contributes to cancer development and metastasis [14]. Primary tumors induce NET formation at foreign sites before metastasis, leading to PMN formation [15]. In addition, NETs can trap disseminating tumor cells, which contributes to early adhesion of tumor cells to foreign sites [16]. NETs shield tumor cells against cytotoxic T lymphocytes and NK cells, thus hampering tumor clearance [17]. NET-DNA can act as a chemotactic factor to attract cancer cells dispersed from their primary site, which is essential for metastasis formation [18]. These studies highlight the important role of NETs in cancer progression, but the mechanisms through which cancer cells modulate NET formation in the PMN have been insufficiently explored.

TLR3, a toll-like receptor, is located inside the cell in endocytic compartments and the endoplasmic reticulum, and recognizes double-stranded RNA (dsRNA) [19] and exosome transfer RNA [20]. Recent studies have underscored the pivotal role of TLR3 in cancer metastasis. Tumor exosomal RNA contributes to host lung epithelial cell TLR3 activation, which is predominantly expressed by alveolar epithelial cells in pre-metastatic lung, inducing neutrophil recruitment and lung metastatic niche formation [21]. Tumor-derived dsRNA induces endothelial slit2 expression, which promotes the migration of cancer cells toward endothelial cells and drives metastatic progression [22].

In this study, we show that metastatic breast cancer cells induce HAO1 expression in alveolar epithelial cells by activating TLR3-IRF3 signaling, rendering an over-production of oxalate. Oxalate accumulation in the lung initiates PMN formation by inducing NETs. In addition, oxalate promotes the proliferation of metastatic cancer cells by activating MAPK signaling. Most importantly, pharmacologic inhibition or knockdown of HAO1 alleviates the pro-metastatic effect of oxalate accumulation.

## Results

### HAO1 is upregulated in alveolar epithelial cells within the lung PMN

To gain insight into metabolic changes induced by primary cancers in the pre-metastatic lung, we analyzed public gene expression profiles of lungs from mice without any tumors and mice bearing low-metastatic (67NR) or high-metastatic (4T1) breast cancer cells in the pre-metastatic phase (GSE62817) [23]. Among the differentially expressed genes, four metabolic-associated genes (HAO1, MGAM, BCHE, and TYMS) were selected for further study (Supplementary Fig. 1A).

**Fig. 1:**
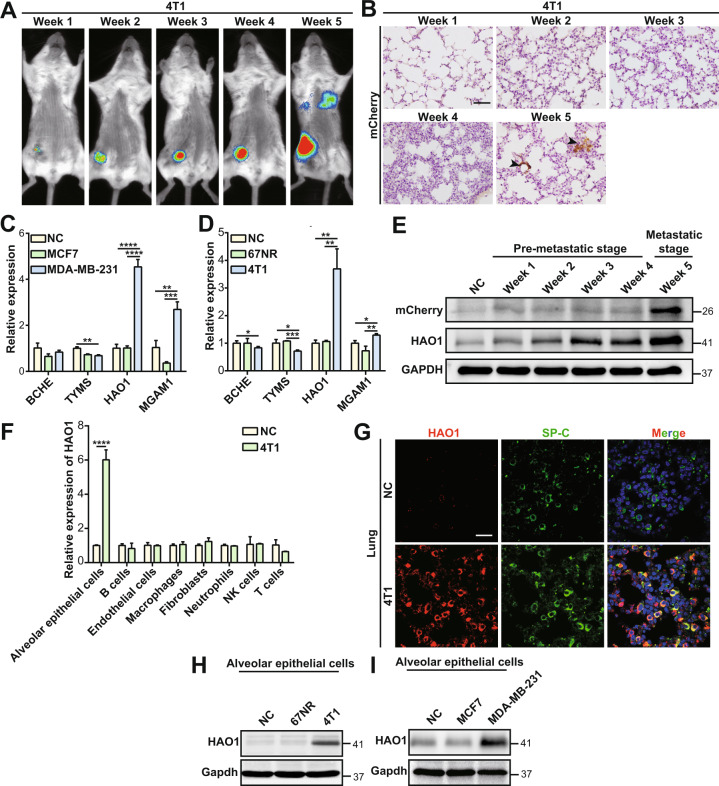
Primary cancer induces pulmonary HAO1 expression. **A** Representative images of bioluminescence imaging of mice bearing luciferase-expressing 4T1 cells for 1–5 weeks. **B** Identification of tumor foci in lungs from mice bearing mCherry-expressing 4T1 cells for 1–5 weeks. The tumor foci were identified by mCherry staining of lung histologic sections. Scale bars, 20 μm. QPCR analysis of HAO1, MGAM, BCHE and TYMS expression in lungs from mice without tumor (blank) and mice bearing MCF7, MDA-MB-231 (**C**), 67NR, 4T1 cells (**D**) for 2 weeks. Means ± s.e.m are provided (*n* = 3). **E** Western blot analysis of mCherry and HAO1 expression in lungs from mice without tumor and mice bearing mCherry-expressing 4T1 cells for 1–5 weeks. **F** QPCR analysis of HAO1 expression in alveolar epithelial cells, B cells, endothelial cells, macrophages, fibroblasts, neutrophils, NK cells and T cells of lung isolated from control mice and mice bearing 4T1 cells for 2 weeks. Means ± s.e.m are provided (*n* = 3). **G** Immunofluorescence staining of HAO1 (red) and SP-C (green) in lungs from naive mice and mice bearing 4T1 cells for 2 weeks. Scale bars, 20 μm. Western blot analysis of HAO1 expression in alveolar epithelial cells cocultured with 67NR, 4T1 (**H**), MCF7 and MDA-MB-231 cells (**I**). *P < 0.05, **P < 0.01, ***P < 0.001, ****P < 0.0001 according to the two-tailed Student’s *t* test.

We performed orthotropic metastasis assay by implanting of several breast cancer cell lines with varied lung metastasis capacities into mice fat pads, including the murine cell lines 67NR (low metastatic potential), 4T1 (high metastatic potential) and human cell lines MCF7 (estrogen receptor+ cells, low metastatic potential), MDA-MB-231 (triple-negative cells, high metastatic potential). Then we monitored lung metastases by applying in vivo bioluminescence imaging, Western blot and immunohistochemistry. The results showed that mice implanted with 4T1 and MDA-MB-231 cells developed lung metastasis after 5 and 7 weeks, respectively (Fig. 1A, B, Supplementary Fig. 1A–D). Thus, we detected the four candidate genes in lung PMNs in the second week after orthotopic implantation of low-metastatic breast cancer cell lines (67NR and MCF7) and high-metastatic cell lines (4T1 and MDA-MB-231). QPCR analyses showed that HAO1 was the most significantly upregulated gene in lung PMN (Fig. 1C, D). Western blot analysis confirmed that HAO1 gradually increased from the first week to the fifth week in mice bearing 4T1 cells (Fig. 1E). Interestingly, we found that orthotopic implantation of metastatic breast cancer cell lines upregulated HAO1 expression only in lung PMNs, but not in brain, bone or liver PMNs (Supplementary Fig. 1E). We then explored whether primary colorectal cancer (CRC) and hepatocellular carcinoma (HCC) could regulate HAO1 expression in the lung PMN. Orthotopic implantation of the CRC cell line CMT93 and HCC cell line HCCLM3 led to the development of lung metastases 6 and 4 weeks later, respectively (Supplementary Fig. 1F, G). Markedly elevated HAO1 expression was observed in the lung PMN at the second week after orthotopic implantation of CMT93 and HCCLM3 cells (Supplementary Fig. 1H, I). Subsequently, we performed fluorescence-activated cell sorting (FACS) to isolate alveolar epithelial cells, B cells, endothelial cells, macrophages, fibroblasts, neutrophils, NK cells and T cells from lungs of normal mice and 4T1-bearing mice. Then we examined the expression of HAO1 in isolated cells by qPCR. The result showed that HAO1 was dramatically upregulated in alveolar epithelial cells from mice inoculated with 4T1 cells compared to that from normal mice (Fig. 1F). Immunofluorescence analysis in lungs from 4T1-bearing mice further corroborated the colocalization of HAO1 and SP-C, a specific marker of alveolar epithelial cells (Fig. 1G). Moreover, we observed a higher expression of HAO1 in alveolar epithelial cells in lung tissue sections from 4T1-bearing mice showed compared with those in mice without tumor (Fig. 1G). We then cocultured alveolar epithelial cells with breast cancer cells in vitro (Supplementary Fig. 1J). Strikingly, alveolar epithelial cells cocultured with metastatic 4T1 and MDA-MB-231cells showed higher expression of HAO1 than the control or those co-cultured with low-metastatic 67NR and MCF7 cells (Fig. 1H, I, Supplementary Fig. 1K, L). These results indicated that HAO1 was upregulated in alveolar epithelial cells at pre-metastatic stage.

### Pharmacologic inhibition or knockdown of HAO1 dampens lung PMN formation and metastasis

**Fig. 2:**
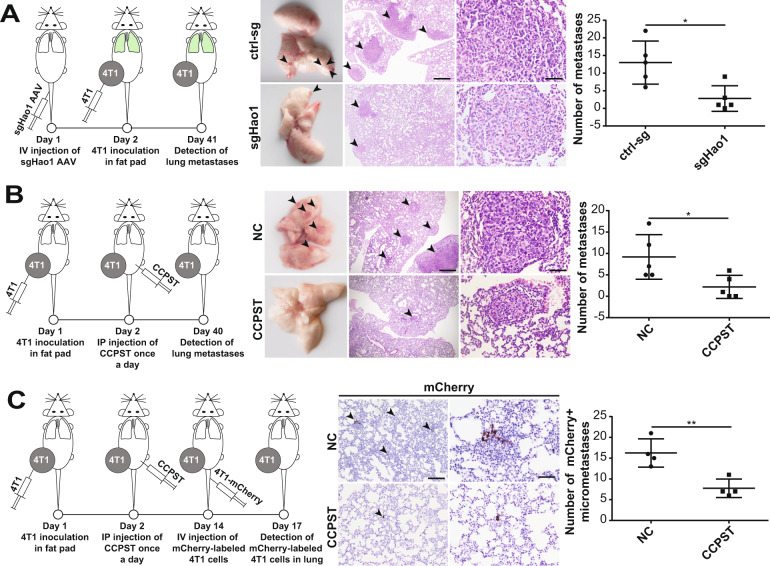
HAO1 induces lung PMN formation. **A** Effect of AAV6-Hao1 sgRNA treatment on lung metastasis in mice bearing 4T1 cells. Means ± s.e.m are provided (*n* = 5). The scale bar in the middle panels represents 200 µm. The scale bar in right panels represents 20 µm. **B** Effect of CCPST treatment on lung metastasis in mice bearing 4T1. Means ± s.e.m are provided (*n* = 5). The scale bar in the middle panels represents 200 µm. Scale bar in the right panels represents 20 µm. **C** MCherry-labeled 4T1 cells were injected into tail vein of mice 2 weeks after inoculation of unlabeled 4T1 cells into fat pad and once daily administration of CCPST. Quantitation of the number of tumor foci identified by mCherry staining of lung histologic sections. Mean ± s.e.m are provided (*n* = 4). The scale bar in the left panels represents 200 µm. The scale bar in the right panels represents 20 µm. *P < 0.05, **P < 0.01 according to two-tailed Student’s *t* test.

To evaluate the role of HAO1 in the formation of lung PMNs and lung metastasis, an AAV6 vector was applied to deliver Hao1 sgRNA to the lungs of the mice before inoculation of 4T1 cells into fat pad. A significant reduction of HAO1 in lung tissue was observed in AAV6-Hao1 sgRNA-infected mice at pre-metastatic stage (Supplementary Fig. 2A, B). The numbers of lung metastases were then detected 40 days after inoculation of 4T1 cells. The result showed that fewer pulmonary metastatic foci were detected in AAV6-Hao1 sgRNA-infected mice (Fig. 2A). 4-carboxy-5-[(4-chlorophenyl)-sulfanyl]-1,2,3-thiadiazole (CCPST) is a specific inhibitor of HAO1 and has been used for substrate reduction therapy in a mouse model of primary hyperoxaluria Type 1 [10]. We thus applied CCPST to block the function of HAO1 in tumor-bearing mice. CCPST was intraperitoneally injected into mice once a day after inoculation of 4T1 cells into the fat pad. Markedly reduction in lung metastases was observed in mice treated with CCPST (Fig. 2B). However, there was no difference in primary tumor growth between the CCPST and control groups (Supplementary Fig. 2C). To further determine whether CCPST hampers cancer-induced PMN formation in the lung, mCherry-expressing 4T1 cells were injected into the tail vein of mice 2 weeks after the inoculation of unlabeled 4T1 cells into the fat pad and once daily administration of CCPST. The results showed that administration of CCPST resulted in a dramatic reduction of mCherry-expressing lung metastatic colonization (Fig. 2C). In addition, we assessed the effect of CCPST on lung metastasis of CRC and HCC, and the results showed that CCPST treatment also attenuated lung metastasis in mice orthotopically implanted with CMT93 and HCCLM3 cells (Supplementary Fig. 2D, E). Collectively, these results illustrated that blockade of HAO1 prevented lung PMN formation and metastasis.

### HAO1 promotes oxalate production in the lung PMN

**Fig. 3:**
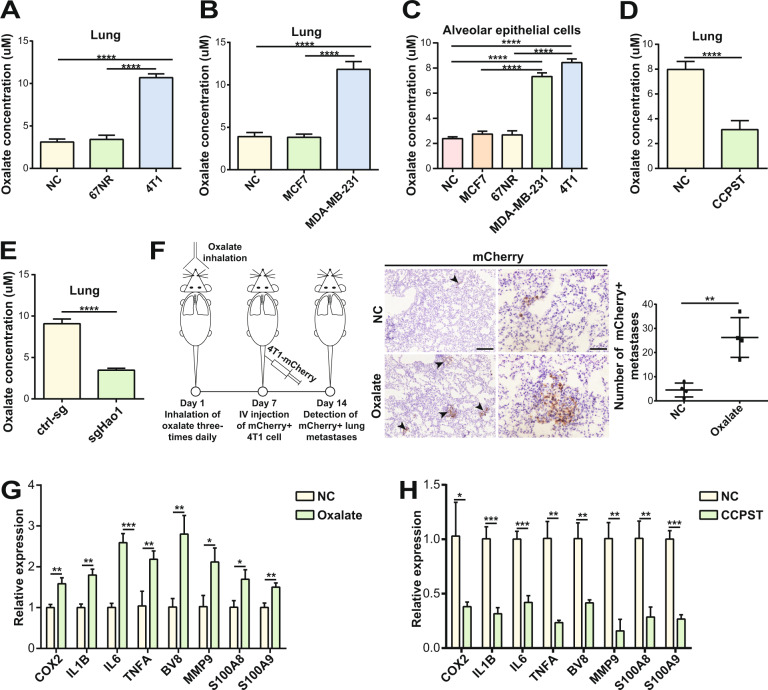
HAO1 promotes oxalate production in the lung PMN. Detection of oxalate concentration in lungs from mice without tumor (NC) and mice bearing 67NR, 4T1 (**A**), MCF7 and MDA-MB-231 cells (**B**) for 2 weeks. Means ± s.e.m are provided (*n* = 3). **C** Detection of the oxalate concentration in alveolar epithelial cells cocultured with 67NR, 4T1, MCF7 and MDA-MB-231 cells. Means ± s.e.m are provided (*n* = 3). **D** Effect of CCPST treatment on pulmonary oxalate production in mice bearing 4T1 cells. Mean ± s.e.m are provided (*n* = 4). **E** Effect of AAV6-Hao1 sgRNA treatment on pulmonary oxalate production in mice bearing 4T1 cells. Means ± s.e.m are provided (*n* = 3). **F** MCherry-labeled 4T1 cells were injected into the tail vein of mice that had inhaled oxalate. The number of tumor foci was determined by mCherry staining of lung tissue sections. Means ± s.e.m are provided (*n* = 4). Scale bar in left panels represents 200 µm. The scale bar in right panels represents 20 µm. **G** QPCR analysis of COX2, IL1B, IL6, TNFA, BV8, MMP9, S100A8 and S100A9 expression in lungs from control mice and mice receiving oxalate inhalation three-times daily for one week. Means ± s.e.m are provided (*n* = 3). **H** QPCR analysis of COX2, IL1B, IL6, TNFA, BV8, MMP9, S100A8 and S100A9 expression in lungs from 4T1-bearing mice receiving once daily administration of CCPST. Means ± s.e.m are provided (*n* = 3). *P < 0.05, **P < 0.01, ***P < 0.001, ****P < 0.0001 according to the two-tailed Student’s *t* test.

HAO1 is a rate-limiting enzyme of oxalate synthesis. Thus we asked whether primary cancer could regulate oxalate production in the lung PMN. Two weeks after orthotopic implantation of breast cancer cells in fat pads, we detected the oxalate concentration in the lungs. The results showed that mice implanted with 4T1 and MDA-MB-231 cells generated higher pulmonary oxalate concentration than those without tumor and those bearing MCF7 and 67NR cells (Fig. 3A, B). In vitro, increased production of oxalate was observed in alveolar epithelial cells cocultured with 4T1 and MDA-MB-231 cells. To confirm that oxalate accumulation induced by primary cancer was HAO1 dependent, we performed a once daily injection of CCPST after orthotopic implantation of 4T1 cells in the fat pad, or injection of sgHao1 AAV via the tail vein the day before orthotopic implantation of 4T1 cells in the fat pad, and then detected the pulmonary oxalate concentration after two weeks. The results showed that both CCPST and HAO1 knockdown suppressed oxalate production in lung PMN induced by primary cancer (Fig. 3D, E).

To ascertain whether oxalate accumulation in lung tissue affected PMN formation and lung colonization of breast cancer cells in vivo, we performed a three-times daily inhalation of oxalate in mice, which led to an elevated oxalate concentration in mice lungs (Supplementary Fig. 3). One week later, mCherry-labeled 4T1 cells were injected via the tail vein into the mice. The results showed that oxalate accumulation promoted lung metastatic colonization compared with the control (Fig. 3F). Oxalate accumulation is capable of inducing inflammation [24]. Thus, we detected the expression of pro-inflammatory factors COX2, IL1B, IL6 and TNFα, pro-metastatic factors BV8, MMP9, S100A8 and S100A9 [21] in the lung from mice inhaled oxalate. As shown in Fig. 3G, oxalate inhalation resulted in marked upregulation of proinflammatory factors and prometastatic factors in mouse lungs. On the contrary, administration of CCPST led to reduced expression of COX2, IL1B, IL6, TNFα, BV8, MMP9, S100A8 and S100A9 in lung of 4T1-bearing mice (Fig. 3H). The above data suggested that HAO1-induced oxalate accumulation was involved in lung PMN formation.

### HAO1-induced oxalate accumulation elicits the formation of NETs

**Fig. 4:**
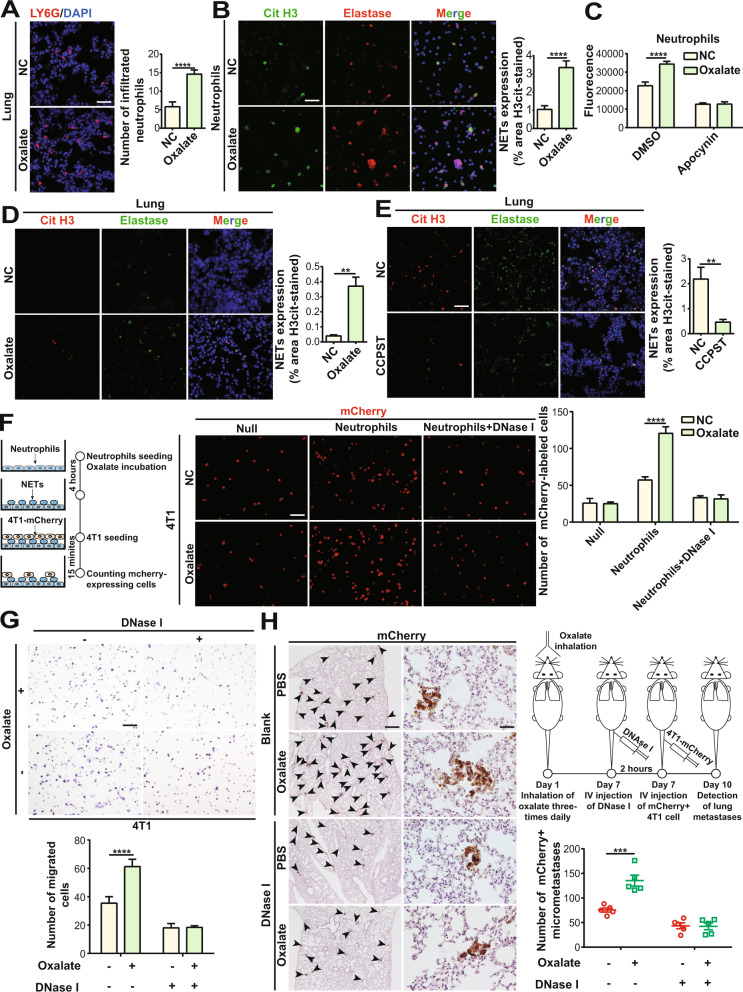
Oxalate accumulation induces NET formation. **A** Images and quantification of neutrophils (labeled by LY6G, red) in the lungs from control mice and mice that received oxalate inhalation three times daily for one week. Scale bars, 20 µm. Means ± s.e.m are provided (*n* = 5). **B** Detection of NETs by immunofluorescence in neutrophils incubated with oxalate. NETs were defined as colocalized elastase (red), citrullinated histone H3 (green), and DNA (blue). Scale bars, 20 µm. Means ± s.e.m are provided (*n* = 5). **C** Detection of ROS production in neutrophils incubated with oxalate. Mean ± s.e.m are provided (*n* = 3). **D** Effect of oxalate inhalation on NET formation in mice lungs. NETs were defined as colocalized citrullinated histone H3 (red), elastase (green), and DNA (blue). Scale bars, 20 µm. Means ± s.e.m are provided (*n* = 5). **E** Effect of CCPST treatment on NET formation in lungs from mice bearing 4T1 cells. Scale bars, 20 µm. Means ± s.e.m are provided (*n* = 5). **F** Effect of PBS, oxalate, PBS + neutrophils, oxalate + neutrophils, PBS + neutrophils + DNase I, oxalate + neutrophils + DNase I treatment on the number of 4T1 cells (labeled by mCherry, red) adhering to the bottom of the wells. Scale bars, 20 µm. Means ± s.e.m are provided (*n* = 5). **G** Images and quantification of 4T1 cells that migrated to NETs derived from neutrophils incubated with oxalate for 4 h. Scale bars, 20 µm. Mean ± s.e.m are provided (*n* = 5). **H** MCherry-labeled 4T1 cells were injected into the tail vein of mice receiving PBS inhalation, oxalate inhalation, PBS inhalation + DNase I injection or oxalate inhalation + DNase I injection. The number of tumor foci was determined by mCherry staining of lung tissue sections. Means ± s.e.m are provided (*n* = 5). The scale bar in left panels represents 200 µm. Scale bar in right panels represents 20 µm. **P < 0.01, ***P < 0.001, ****P < 0.0001 according to the two-tailed Student’s *t* test.

We next examined the changes in inflammatory cellular composition within lung PMNs induced by oxalate accumulation. We analyzed the number of many kinds of inflammatory cells including macrophages, neutrophils, B lymphocytes and T lymphocytes, in lung tissue from mice that inhaled oxalate. Immunofluorescence analyses confirmed that aerosol inhalation of oxalate in mice led to significant neutrophil recruitment (Fig. 4A, Supplementary Fig. 4A–D). In contrast, CCPST treatment dramatically reduced the number of pulmonary neutrophils in 4T1-bearing mice (Supplementary Fig. 4E). Previous studies have suggested that oxalate induces overproduction of ROS by activating NADPH oxidase [4]. ROS production in neutrophils induced by the activation of the NADPH oxidase is considered an initial step in NET formation [13], which plays an important role in cancer metastasis. Thus, we postulated that NETs might contribute to oxalate-induced PMN formation in the lung. To confirm this hypothesis, neutrophils were isolated from the bone marrow of mice (Supplementary Fig. 4F) and treated with oxalate in vitro. Immunofluorescence staining showed that oxalate treatment led to NET formation (Fig. 4B). Increased ROS production in neutrophils was also observed under the oxalate treatment. However, apocynin, an inhibitor of NADPH oxidase, effectively reduced ROS production in neutrophils stimulated by oxalate (Fig. 4C). We also explored the effect of oxalate accumulation on the formation of NETs in vivo. The results showed that the accumulation of oxalate in lung tissue induced NET formation (Fig. 4D). Conversely, CCPST treatment suppressed pulmonary NET formation in 4T1-bearing mice (Fig. 4E). These results demonstrated that HAO1-induced oxalate accumulation contributes to NET formation in the lung PMN.

NET-mediated cell trapping facilitates early adhesion of cancer cells to foreign sites [16]. Thus, we examined whether oxalate-induced NET formation could promote the adhesion of cancer cells. The results showed that exposure of neutrophils to oxalate was sufficient to increase adhesion of breast cancer cells in vitro. However, this effect was significantly alleviated by the addition of DNase I to scavenge NETs (Fig. 4F, Supplementary Fig. 5A–C). It has been reported that NET-DNA acts as a chemotactic factor to attract disseminating cancer cells [18]. Our results showed that NETs derived from oxalate-treated neutrophils recruited more 4T1 cells, while digesting NET DNA with DNase I attenuated this effect (Fig. 4G). Subsequently, we investigated the role of NETs in oxalate-induced lung PMN formation. DNase I was injected intravenously into mice that inhaled oxalate to degrade NETs. Two hours later, 4T1 cells were injected into the mice via the tail vein and lung metastases were detected. Obviously, administration of DNase I abolished the pro-metastatic effect of oxalate (Fig. 4H). Taken together, these data demonstrated that HAO1-induced oxalate accumulation led to NET formation in the lung PMN.

### Oxalate accumulation promotes the proliferation of metastatic cancer cells by activating MAPK signaling

**Fig. 5:**
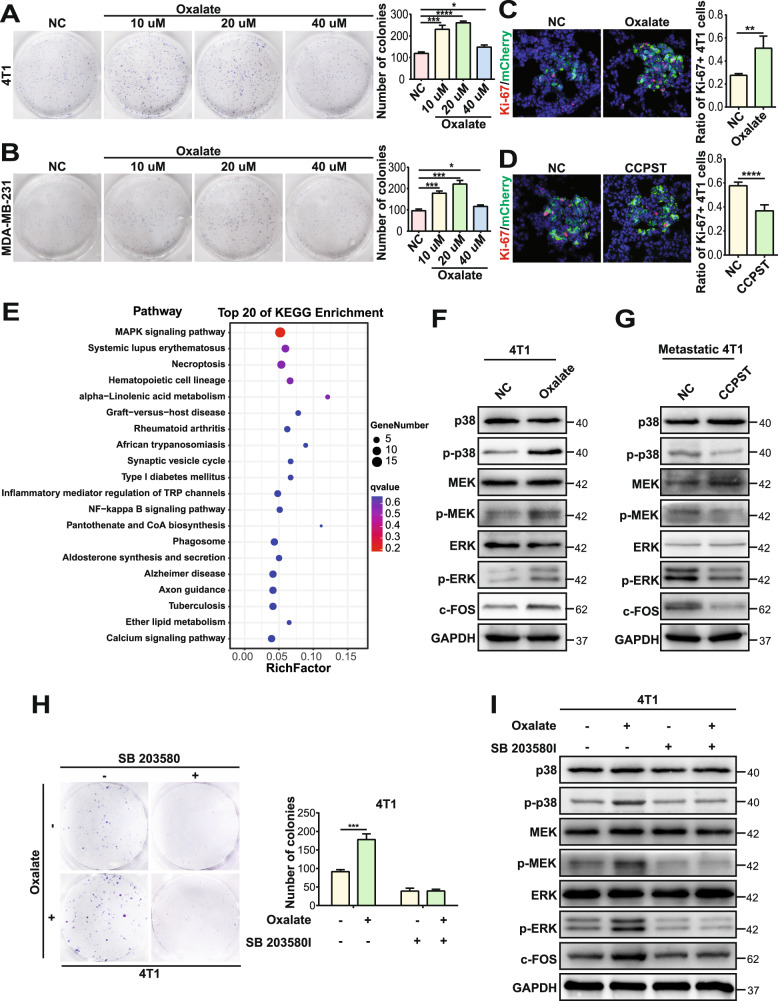
Oxalate accumulation promotes the proliferation of metastatic cancer cells. Effect of oxalate treatment on the proliferation of 4T1 (**A**) and MDA-MB-231 cells (**B**). Means ± s.e.m are provided (*n* = 3). **C** Ratio of Ki-67 + 4T1 cells (red)/total 4T1 cells (labeled by mCherry, green) in lungs from mice inhaled oxalate. Means ± s.e.m are provided (*n* = 5). Scale bar, 20 µm. **D** Ratio of Ki-67 + 4T1 cells (red)/total 4T1 cells (labeled by mCherry, green) in lungs from mice receiving a once daily administration of CCPST. Means ± s.e.m are provided (*n* = 5). Scale bar, 20 µm. **E** KEGG pathway enrichment analysis of differentially expressed genes between 4T1 cells incubated with PBS or oxalate. **F** Effect of oxalate treatment on p38, p-p38, MEK, p-MEK, ERK, p-ERK and c-fos expression in 4T1 cells. **G** Effect of CCPST treatment on p38, p-p38, MEK, p-MEK, ERK, p-ERK and c-fos expression in metastatic 4T1 cells isolated from lungs of tumor-bearing mice. **H** Changes in the growth rate in 4T1 treated with PBS, oxalate, SB 203580 + PBS or SB 203580 + oxalate. Means ± s.e.m are provided (*n* = 3). **I** Changes in p38, p-p38, MEK, p-MEK, ERK, p-ERK and c-fos expression in 4T1 treated with PBS, oxalate, SB 203580 + PBS or SB 203580 + oxalate. *P < 0.05, **P < 0.01, ***P < 0.001, ****P < 0.0001 according to the two-tailed Student’s *t* test.

A previous study has suggested that oxalate treatment promotes breast cancer growth [6]. We detected the proliferation of 4T1 and MDA-MB-231 cells under treatment with different doses of oxalate in vitro. The results of the colony formation assay showed that oxalate could stimulate the proliferation of breast cancer cells, especially at a concentration of 20 µM (Fig. 5A, B). Metastatic lesions of 4T1 cells were detected in the lungs of mice that inhaled oxalate and showed enhanced proliferation (shown using Ki-67) (Fig. 5C). However, CCPST administration dampened the proliferation of metastatic lesions in the lung of 4T1-bearing mice (Fig. 5D). These results suggested that exposure of breast cancer cells to oxalate promoted cancer outgrowth. To reveal the potential mechanisms by which oxalate induced breast cancer cell proliferation, we performed RNA-Seq to screen the differentially expressed genes in 4T1 cells treated with oxalate. We identified 959 differentially expressed genes and found that genes upregulated in oxalate-treated 4T1 cells were associated with the MAPK signaling pathway (Fig. 5E). Western blot analysis confirmed that oxalate induced p-p38, p-MEK, p-ERK and c-fos expression in 4T1 cells, suggesting that oxalate activated MAPK signaling pathway (Fig. 5F). We then isolated mCherry-expressing 4T1 cells from the lungs of 4T1-bearing mice treated with CCPST by FACS. The expression of p-p38, p-MEK, p-ERK and c-fos expression in isolated cells were detected by Western blot. The result showed that CCPST administration led to MAPK signaling pathway inactivation in pulmonary metastatic 4T1 cells (Fig. 5G). Next, SB 203580, an inhibitor of p38 MAPK, was applied to inactivate the MAPK signaling pathway in 4T1 cells. The results of the colony formation assay showed that SB 203580 treatment markedly suppressed the pro-proliferative effect of oxalate on 4T1 cells (Fig. 5H). Moreover, Western blot analysis confirmed that SB 203580 treatment abolished oxalate-induced upregulation of p-38, p-MEK, p-ERK and c-fos (Fig. 5I). These results indicated that oxalate accumulation promoted the proliferation of metastatic cancer cells by activating the MAPK signaling pathway.

### Primary cancer induces HAO1 expression in alveolar epithelial cells by activating TLR3-IRF3 signaling

**Fig. 6:**
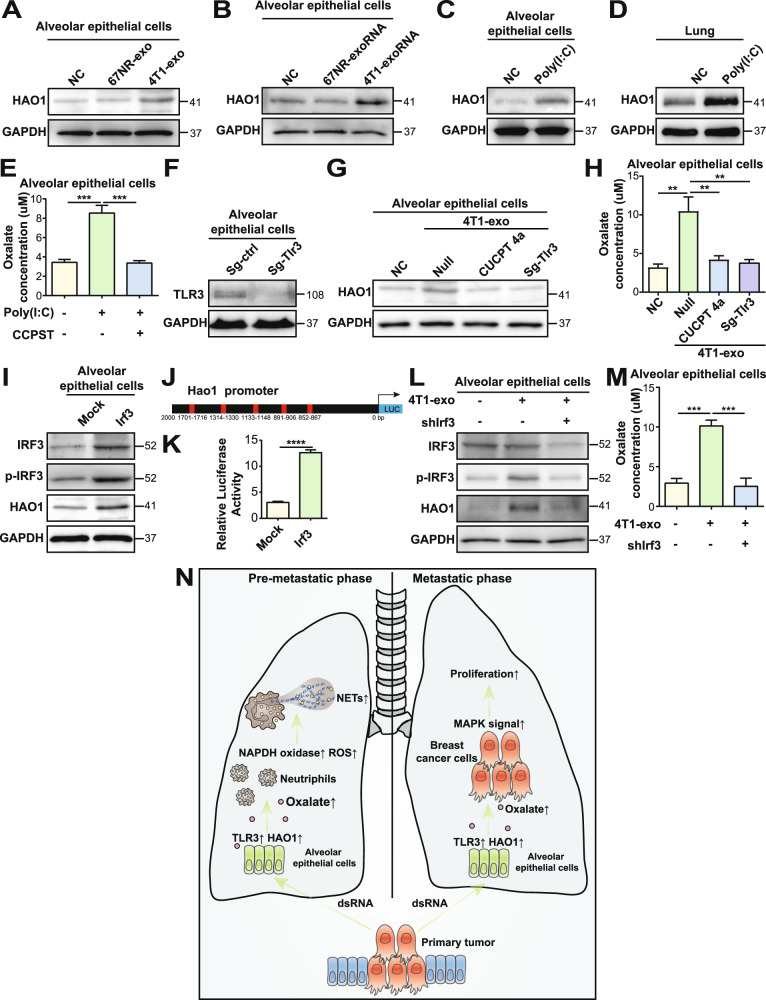
TLR3 signal activation induces HAO1 expression in alveolar epithelial cells. **A** Effect of 67NR and 4T1 exosomes treatment on HAO1 expression in alveolar epithelial cells by Western blot analysis. **B** Effect of transfection of RNA isolated from 67NR and 4T1 exosomes on HAO1 expression in alveolar epithelial cells by Western blot analysis. **C** Effect of poly (I:C) treatment on HAO1 expression in alveolar epithelial cells by Western blot analysis. **D** Effect of poly (I:C) inhalation on HAO1 expression in mice lung by Western blot analysis. **E** Detection of oxalate production in alveolar epithelial cells treated with poly(I:C) or poly(I:C) + CCPST. Means ± s.e.m are provided (*n* = 3). **F** Detection of TLR3 expression in alveolar epithelial cells infected with sg-control or sg-Tlr3 lentivirus. **G** Detection of HAO1 expression in alveolar epithelial cells treated with 4T1 exosomes, 4T1 exosomes + CU CPT 4a or 4T1 exosomes + sg-Tlr3 lentivirus by Western blot analysis. **H** Detection of oxalate production in alveolar epithelial cells treated with 4T1 exosomes, 4T1 exosomes + CU CPT 4a or 4T1 exosomes + sg-Tlr3 lentivirus. **I** Effect of IRF3 over-expression on IRF3, P-IRF3 and HAO1 expression in alveolar epithelial cells by Western blot analysis. **J** Diagram of the HAO1 promoter showing the location of IRF3 binding sites. **K** Luciferase activity of HAO1-promoter construct after transfection of IRF3 plasmid in alveolar epithelial cells. **L** Detection of IRF3, P-IRF3 and HAO1 expression in alveolar epithelial cells treated with 4T1 exosomes or 4T1 exosomes + shIRF3 by Western blot analysis. **M** Detection of oxalate production in alveolar epithelial cells treated with 4T1 exosomes or 4T1 exosomes + shIRF3. Means ± s.e.m are provided (*n* = 3). **N** Schematic diagram of the role of HAO1-mediated oxalate metabolism at pre-metastatic stage and metastatic stage. ***P < 0.001, ****P < 0.0001 according to two-tailed Student’s *t* test.

Finally, we explored the mechanism by which primary cancer induces HAO1 expression in alveolar epithelial cells. Primary tumors can induce PMN formation at foreign sites by releasing exosomes, which are defined as lipid-bilayer vesicles containing protein, RNA, and DNA [1, 25, 26]. To examine whether exosomes were involved in tumor-induced HAO1 expression, we derived exosomes from 67NR and 4T1 cells for further study (Supplementary Fig. 6A, B). Apparently, exosomes from 4T1 cells, but not those from 67NR, led to increased HAO1 expression in alveolar epithelial cells (Fig. 6A). Moreover, we also observed elevated HAO1 expression in alveolar epithelial cells transfected with RNA isolated from 4T1-derived exosomes (Fig. 6B). A previous study has shown that the TLR3 pathway is one of the most significantly altered pathways in alveolar epithelial cells at pre-metastatic lung. Activation of TLR3 in alveolar epithelial cells is critical for initiating neutrophil recruitment and lung metastatic niche formation by sensing tumor exosomal double-stranded RNA (dsRNA) [21]. We asked whether TLR3 was involved in the regulation of HAO1 expression in alveolar epithelial cells. We treated alveolar epithelial cells with poly (I:C), an agonist of TLR3. Western blot and qPCR analysis showed that the expression of HAO1 in alveolar epithelial cells was significantly increased by poly(I:C) treatment in vitro (Fig. 6C, Supplementary Fig. 6C). Congruently, inhalation of poly(I:C) in mice resulted in a significant upregulation of HAO1 in lung (Fig. 6D, Supplementary Fig. 6D). Moreover, poly(I:C) incubation promoted oxalate production in alveolar epithelial cells, while CCPST treatment reversed this effect (Fig. 6E). Then, we pharmacologically inhibited that function of TLR3 by using CU CPT 4a or knocked out TLR3 by applying CRISPR/Cas9 gene-editing strategy in alveolar epithelial cells (Fig. 6F). The results of Western blot showed that pharmacological inhibition or knock-out of TLR3 abolished 4T1 exosome-induced HAO1 expression and oxalate production in alveolar epithelial cells (Fig. 6G, H).

To investigate the mechanism through which TLR3 modulates HAO1 expression, we overexpressed IRF3, p-65 and JUN, which are classic downstream of TLR3, in alveolar epithelial cells. The results showed that overexpression of IRF3, but not p-65 or JUN, dramatically induced HAO1 expression in alveolar epithelial cells (Fig. 6I, Supplementary Fig. 6E–G). Then, we analyzed transcription factors located within a 2-kb region directly upstream of the transcription start site of HAO1 using the TFtarget database and found several binding motifs for IRF3 (Fig. 6J). A luciferase reporter assay showed that over-expression of IRF3 led to elevated luciferase activity of the HAO1 promoter in alveolar epithelial cells (Fig. 6K). These results verified that IRF3 increased the promoter activity of HAO1. We then knocked down IRF3 expression in alveolar epithelial cells treated with poly(I:C) or incubated with 4T1-derived exosomes. Western blot analysis suggested that IRF3 knockdown hampered HAO1 expression induced by poly(I:C) or 4T1-derived exosomes (Fig. 6L, Supplementary Fig. 6H). Moreover, IRF3 knockdown resulted in a dramatic reduction of oxalate in alveolar epithelial cells treated with poly(I:C) or incubated with 4T1-derived exosomes (Fig. 6M, Supplementary Fig. 6I). These findings illustrated that TLR3-IRF3 signaling was necessary for HAO1 expression and oxalate production in alveolar epithelial cells induced by cancer cells.

## Discussion

Cancer cells can not only adapt their metabolism during tumorigenesis [27], but also modulate the metabolic reprogramming of the PMN to promote tumor metastasis [1, 2, 28]. Reprogramming of glucose metabolism in recipient PMN cells promotes the development of metastasis [2]. However, the impact of the enriched metabolic components in PMNs on tumor metastasis have been insufficiently explored. In this study, the capacity of HAO1-induced oxalate metabolism to promote the formation of lung PMNs was demonstrated for the first time.

Oxalate is a potentially toxic dicarboxylic acid that is not further metabolized by mammals [3]. Oxalate has been shown to be a tumor promoting factor in breast cancer [6], but its role in cancer progression has not been reported. HAO1 is the key enzyme in the pathway of oxalate production and has the ability to oxidize glycolate to glyoxylate and glyoxylate to oxalate [7, 8, 10]. Suppression of HAO1 has been shown in preclinical models to prevent the formation of calcium oxalate [10, 12, 29]. In this study, we found that metastatic breast cancer elevated HAO1 expression in lung alveolar epithelial cells, which led to oxalate accumulation in the lung PMN. Alveolar epithelial cells not only serve as a biological barrier in the respiratory tract, but also play an essential role in the recognition of injury-associated signals, orchestrating innate immunity in the lung [30]. Oxalate accumulation promoted the release of many pro-inflammatory and pro-metastatic factors in lung tissue and ultimately lung metastatic colonization. Then, we evaluated whether inhibition of HAO1 could be used to prevent lung metastasis of cancer. Administration of CCPST, a HAO1 inhibitor, has been previously described as a feasible strategy to reduce oxalate production both in vivo and in vitro [10]. Our data showed that CCPST and AAV6-Hao1 sgRNA suppressed oxalate production in lung PMNs induced by metastatic breast cancer and lung metastasis. These results suggest that HAO1 is an eligible therapeutic target to prevent PMN-promoted lung metastasis.

Recent studies have focused on oxalate as an activator of inflammatory pathways [24]. We investigated the effect of HAO1-induced oxalate accumulation on the inflammatory response within lung PMN. We observed the significantly greatest aggregation of neutrophils in lung of mice that inhaied oxalate. The recruitment of neutrophils is considered an essential event in cancer-induced PMN formation [31]. A previous study has revealed that oxalate induces oxidative stress by the production of ROS via NADPH oxidase [4]. In neutrophils, NET formation depends on the generation of ROS by NADPH oxidase [13], which promotes cancer metastasis by trapping disseminating tumor cells and activating the CCDC25-ILK-β-parvin-RAC1-CDC42 cascade in cancer [16, 18]. Hence, we assumed that oxalate accumulation within the lung PMN might contribute to metastasis by inducing NET formation. As expected, our results showed that HAO1-induced oxalate accumulation promoted NET formation in the lung PMN and attracted disseminating cancer cells. However, degradation of NETs by DNase I hampered the pro-metastatic effect of oxalate and inhibited lung metastatic colonization in mice.

It has been reported that chronic exposure of breast epithelial cells to oxalate promotes the transformation of breast cells from normal to tumor cells, inducing the expression of proto-oncogenes such as c-fos and proliferation in breast cancer cells [6]. We also confirmed that oxalate accumulation promoted the growth of breast cancer in vitro and in vivo. RNA-Seq analyses showed that HAO1-induced oxalate accumulation enhanced the proliferation of breast cancer cells mainly by activating MAPK signaling. MAPK signaling regulates the expression of genes involved in many processes that play a key role in the development and progression of cancer, such as proliferation, migration and apoptosis [32]. We found that inhibition of the MAPK signaling pathway markedly suppressed the proliferation of cancer cells induced by oxalate.

Exosomes derived from primary tumors are critical for PMN formation in distant organs [21, 25, 26]. We found that stimulation of 4T1-derived exosomes or transfection of exosomal RNA from 4T1 cells was sufficient to induce HAO1 expression in alveolar epithelial cells. A recent study has shown that tumor exosomal RNAs promote lung PMN formation by activating TLR3, which is predominantly expressed by alveolar epithelial cells in pre-metastatic lungs [21]. TLRs can recognize exogenous and endogenous stimulators to prime immune responses [33]. As sensors for dsRNA, tumor TLR3 promotes tumor cell survival, proliferation and chemotherapy resistance [34], or improves sensitivity to poly(I:C)-mediated cancer therapy [35]. We investigated the role of TLR3 signaling in the upregulation of HAO1 in alveolar epithelial cells and found that TLR3 signaling was required for HAO1 expression in alveolar epithelial cells induced by primary breast cancer. High expression of lung epithelial TLR3 is related to smoke-induced chronic inflammation [36]. We also found that pharmacological inhibition of TLR3 abolished breast cancer-induced HAO1 expression and oxalate production. Moreover, our results verified that IRF3, the major transcription factor activated by TLR3, increased promoter activity of HAO1. Knockdown of IRF3 dampened HAO1 expression and oxalate production induced by poly(I:C) or 4T1-derived exosomes. Thus, activation of TLR3-IRF3 signaling promotes HAO1-induced oxalate accumulation in alveolar epithelial cells.

In summary, we unveiled an oxalate metabolism-dependent mechanism for lung PMN formation and metastasis (Fig. 6N). Targeting HAO1-mediated oxalate metabolism is a feasible approach to prevent and treat cancer metastasis to the lung.

## Methods

No sample size calculations were performed beforehand, but all experiments were repeated at least three times. Sample size was determined according to standard practices of field of Cell biology. No samples were excluded from the analysis. Randomization is not relevant to the experiments performed in this study because randomization of samples is not applicable to cell lines and in vitro studies. No experiment has been blinded. The variance between the groups is similar.

### Cell culture and treatment

Human cancer cell lines MCF7, MDA-MB-231, HCCLM3 and murine cancer cell lines 67NR, 4T1, CMT93 were obtained from ATCC. All cells were cultured in Dulbecco’s modified Eagle’s medium (DMEM; Gibco) with 10% fetal bovine serum Gibco), 1% penicillin and streptomycin, and they were maintained at 37 °C and 5% CO_2_. MCherry and luciferase-expressing 4T1 cells were established by transfection with lentivirus expressing mCherry and luciferase. The sequences of sgRNA and shRNA referred above were listed in Supplementary Tables 1–3. The potassium oxalate (20 μM, Aladdin), DNase I (100 U/ml, Aladdin), CCPST (200 μM, Key organics), PMA (10 nM, MedChemExpress), apocynin (10 μM, MedChemExpress), SB 203580 (500 nM, MedChemExpress) and CU CPT 4a (27 μM, Tocris) were used for cell treatment.

### Isolation of neutrophils and alveolar epithelial cells

For isolation of neutrophils, bone marrow cells derived from 6 to 8-week-old BALB/c mice were harvested in Hank’s buffered salt solution (HBSS; Gibco), subsequently filtered through 40-μm nylon mesh and added to the top of a 2-layer Percoll (GE Healthcare) gradient (72% and 63.5% in PBS), followed by centrifugation at 810 g for 20 min at 4 °C. Neutrophils enriched in the interface of 63.5–72% fractions were confirmed to be of >95% purity by immunofluorescence (Supplementary Fig. S4F). For isolation of alveolar epithelial cells, BALB/c mice were perfused with 5 ml of HBSS through the pulmonary artery via the right ventricle and instilled with 4.5 U/ml of elastase (Roche Diagnostics) via tracheal cannula. The lung lobes were then minced in 100 U/ml DNase I (Aladdin). Cells in suspension were subsequently filtered through 40 μm nylon mesh and then enriched by IgG panning. The purified alveolar epithelial cells were confirmed to be of >90% purity by immunofluorescence (Supplementary Fig. 1J).

### Mice and tumor models

The sample size for animal studies was determined according to standard practices of field of Cell biology. Wherever quantification is provided, at least 3 independent experiments were carried out to perform statistical analysis. Allocation of animals to the different groups was random by trained researchers performing each experiment. 6 to 8-week-old female BALB/c, C57BL/6 and athymic mice were used in all animal experiments. All protocols for the animal studies were approved by the Institutional Animal Care and Use Committee of Southern Medical University. For the orthotropic metastasis assay of breast cancer, MCF7, MDA-MB-231 (1 × 10^6^ cells) were injected into the fourth mammary fat pad of athymic mice, and 67NR (1 × 10^6^ cells) and 4T1 cells (1 × 10^5^ cells) were injected into the fourth mammary fat pad of BALB/c mice. For the orthotropic metastasis assay of CRC, CMT93 cells (1 × 10^6^ cells) were injected into the mesentery at the tail end of the cecum of C57BL/6 mice. For the orthotropic metastasis assay of HCC, HCCLM3 cells (1 × 10^6^ cells) were injected into the liver of athymic mice. For the tail vein metastasis assay, 5 × 10^5^ 4T1 cells were injected into the tail vein of mice. For oxalate or poly(I:C) administration, mice inhaled potassium oxalate (20 mg/ml) three-times daily for 20 min or once daily with exposure to poly(I:C) (2 mg/ml) for 20 min. For CCPST administration, mice received an intraperitoneal injection of CCPST (10 mg/kg) once daily. For AAV administration, mice were injected with 10^11^ vg AAV6-control sgRNA or AAV6-Hao1 sgRNA (expressing HA-tag, GeneCopoeia, the sequences are shown in Supplementary Table 1) diluted in 100 μl PBS, respectively, via the tail vein.

### Western blot

Tissue and cells were lysed with RIPA buffer (Fdbio science) and quantified using the Bradford Protein Assay (KeyGEN BioTECH). Equal amounts of extracts were subjected to SDS–PAGE, and transferred onto nitrocellulose membranes (Millipore). Then, the membranes were incubated with primary antibodies overnight at 4 °C. The following primary antibodies were used: HAO1 (Immunoway, YM3378, 1:1000 dilution), GAPDH (Proteintech, 60004-1-Ig, 1:5000 dilution), mCherry (Abcam, ab213511, 1:2000 dilution), TSG101 (Proteintech, 14497-1-AP, 1:1000 dilution), IRF3 (Proteintech, 11312-1-AP, 1:1000 dilution), p-IRF3 (Affinity, AF3438, 1:1000 dilution), TLR3 (Novus Biologicals, IMG-315A, 1:1000 dilution), p65 (Cell signaling technology, # 8242, 1:1000 dilution), p-p65 (Cell signaling technology, # 3033, 1:1000 dilution), JUN (Cell signaling technology, # 9165, 1:1000 dilution), p-JUN (Cell signaling technology, #91952, 1:1000 dilution), MEK (Cell signaling technology, # 4694, 1:1000 dilution), p-MEK (Cell signaling technology, #9152, 1:1000 dilution), ERK (Cell signaling technology, # 4695, 1:1000 dilution), p-ERK (Cell signaling technology, # 4370, 1:1000 dilution), C-FOS (Cell signaling technology, # 2250, 1:1000 dilution), p38 (Cell signaling technology, #8690, 1:1000 dilution) and p-p38 (Cell signaling technology, #4511, 1:1000 dilution). Following incubation with the HRP-conjugated antibody (Fdbio science, FDM007 or FDR007, 1:10000 dilution). Bands were detected using FDbio-Femto ECL Western blotting detection reagents (Fdbio science).

### QPCR and RNA sequencing

Total RNA was extracted from tissue and cells using TRIzol reagent (Clontech Laboratories) according to the manufacturer’s instructions. Reverse transcription was performed using PrimeScript™ RT Master Mix (Clontech Laboratories). Quantitative real-time PCR analysis was performed using SYBR Green PCR Master Mix (TaKaRa). The sequences of all indicated primers are listed in Supplementary Table 4. For RNA sequencing, the enriched mRNA from 4T1 cells treated with or without potassium oxalate (20 μM) was reverse transcribed into cDNA. The cDNA libraries were sequenced on the Illumina sequencing platform by Genedenovo Biotechnology Co., Ltd (Guangzhou, China). The data are deposited at the Gene Expression Omnibus (accession number: GSE166167).

### Immunofluorescence

Paraffin-embedded tissue blocks were cut into 2.5-μm sections and transferred to glass slides. Sections were immersed in 3% hydrogen peroxide and incubated with primary antibodies overnight at 4 °C. Subsequently, sections were stained with goat anti-mouse and anti-rabbit IgG/Alexa Fluor (Bioss Antibodies). The following primary antibodies were used: HAO1 (Immunoway, YM3378, 1:200 dilution), SP-C (Proteintech, 10774-1-AP, 1:200 dilution), mCherry (Abcam, ab213511, 1:500 dilution), citrullinated histone H3 (Abcam, ab5103, 1:200 dilution), neutrophil elastase (Abcam, ab131260, 1:200 dilution).

### Fluorescence-activated cell sorting

BALB/c mice were perfused with 5 ml of HBSS through the pulmonary artery via the right ventricle and instilled with 4.5 U/ml of elastase (Roche Diagnostics) via tracheal cannula. The lung lobes were then minced in 100 U/ml DNase I (Aladdin). Cells in suspension were subsequently filtered through a 40 μm nylon mesh and then sorted by FACS. Anti-CD45 (BioLegend, 157607, 1:200 dilution) and anti-CD3 (BioLegend, 100209, 1:200 dilution) antibodies were used for the isolation of T cells. Anti-CD45 and anti-CD19 (BioLegend, 152407, 1:200 dilution) antibodi were used for isolation of B cells. Anti-CD45 and anti-NK-1.1 (BioLegend, 108703, 1:200 dilution) antibody were used for isolation of NK cells. Anti-CD45 and anti-F4/80 (BioLegend, 123105, 1:200 dilution) antibodies were used for the isolation of macrophages. Anti-CD45 and anti-Ly6G (BioLegend, 127627, 1:200 dilution) antibodies were used for the isolation of neutrophils. Anti-EpCAM (BioLegend, 324215, 1:200 dilution) and anti-SP-C (abclonal, A1835, 1:500 dilution) antibodies were used for the isolation of alveolar epithelial cells. Anti-CD31 (BioLegend, 102409, 1:200 dilution) antibody was used for the isolation of endothelial cells. Anti-S100A4 (abcam, ab197896, 1:500 dilution) antibody was used for the isolation of fibroblasts.

### Determination of the oxalate concentration in lungs and cells

oxalate concentration in lungs and cells were determined using an oxalate assay kit (Biovision, K663). A total of 5 mg lung tissue or 5 × 10^5^ cells was ground and resuspended in 100 µl Oxalate Assay Buffer, following by a centrifugation at 12,000 rpm for 10 min. The supernatant was collected for oxalate detection. The reaction mix was added and incubated at 37 °C for 30 min. The OD value at 450 nm was measured.

### Colony formation assay

A total of 500 cells were plated in each well of 6-well plates and were cultured in DMEM with 2% fetal bovine serum. The medium was renewed every 3 days. After 10 days, the cells were fixed using 4% paraformaldehyde for 30 min, stained with hematoxylin for 30 min and then photographed.

### Purification of NETs and transwell migration assay

Neutrophils were treated with 20 nM PMA or 20 nM PMA plus 20 μm potassium oxalate for 4 h. NETs adhering to the bottom of the wells were then washed with DMEM containing 2% fetal bovine serum and were centrifuged at 1000 × *g* at 4 °C for 10 min. The supernatant containing NETs was collected. Subsequently, 5 × 10^4^ cells were suspended in serum-free DMEM and seeded into the transwell chambers with 8-μm-pore-size inserts (BD Biosciences). The medium containing NETs with or without DNase I (100 U/ml) was placed into the bottom chamber. After 6 h, the cells that had migrated through the membrane and stuck to the lower surface of the membrane and were counted as cells per field of view under light microscopy.

### Adherence assay

A total of 5 × 10^5^ neutrophils were plated in 24-well plates and treated with 20 nM PMA, 20 nM PMA plus 20 µm potassium oxalate or 20 nM PMA plus 20 μm potassium oxalate and 100 U/ml DNase I for 4 h. MCherry-expressing 4T1 cells (1 × 10^5^) were added to each well and were allowed to adhere to the plate bottom for 15 min. Subsequently, the wells were fixed using 4% PFA. Adherent cells were stained with anti-mCherry antibody, and the number of mCherry-expressing 4T1 cells was counted as cells per field of view under confocal microscopy.

### NET formation assay

Neutrophils were treated with 20 nM PMA or 20 nM PMA plus 20 μm potassium oxalate for 4 h. Then, the neutrophils were fixed with 4% PFA and than stained with anti-elastase and anti-citrullinated histone H3. NET formation was determined as the percentage of the field of view positive for a citrullinated histone H3.

### ROS analysis

Neutrophils were treated with 20 nM PMA, 20 nM PMA plus 20 μm potassium oxalate or 20 nM PMA plus 20 μm potassium oxalate and 10 μM apocynin for 2 h. Subsequently, the cells were incubated with 10 mM CM-H2DCFDA (Yeasen), for 15 min and then washed with pre-chilled PBS, and the fluorescence of each well was monitored at 480 nm excitation and 540 nm emission.

### Isolation, identification and treatment of exosomes

Exosomes were derived from breast cancer cell conditioned media by ultracentrifugation. Breast cancer cells were cultured in DMEM medium with 10% fetal bovine serum, which was depleted of exosomes by ultracentrifugation at 120,000 × *g* for 8 h at 4 °C prior to use. Conditioned media were centrifuged at 500 × *g* for 10 min at 4 °C, followed by 16,800 × *g* for 30 min at 4 °C. The supernatants were passed through a 0.22-μm filter (Millipore) and ultracentrifuged at 120,000 × *g* for 70 min at 4 °C. The exosomes were washed with PBS and then resuspended in PBS. The amount of exosomes was determined by the Bradford Protein Assay (KeyGEN BioTECH). Transmission electron microscopy was performed as as previously described [37]. For cell treatment, a total of 5 µg exosomes were incubated with 5 × 10^5^ recipient cells for 48 h.

## Supplementary information

Supplementary Information

## Data Availability

The RNA sequencing data from 4T1 cells treated with or without potassium oxalate (20 μM) are deposited at Gene Expression Omnibus (accession number: GSE166167). All other remaining data are included in the article and Supplementary Information files, or available from the authors upon reasonable request.
