# Supplementary material for: HAO1-mediated oxalate metabolism promotes lung pre-metastatic niche formation by inducing neutrophil extracellular traps
Source: Oncogene. 2022 Jun 23;41(29):3719–31. doi: 10.1038/s41388-022-02248-3 (PMC9287177; doi:10.1038/s41388-022-02248-3)
Supplement: Supplementary file 1 — Supplementary Information [file 41388_2022_2248_MOESM1_ESM.docx]

**HAO1-mediated Oxalate Metabolism Promotes Lung Pre-metastatic Niche Formation by Inducing Neutrophil Extracellular Traps**

Zeng et al.


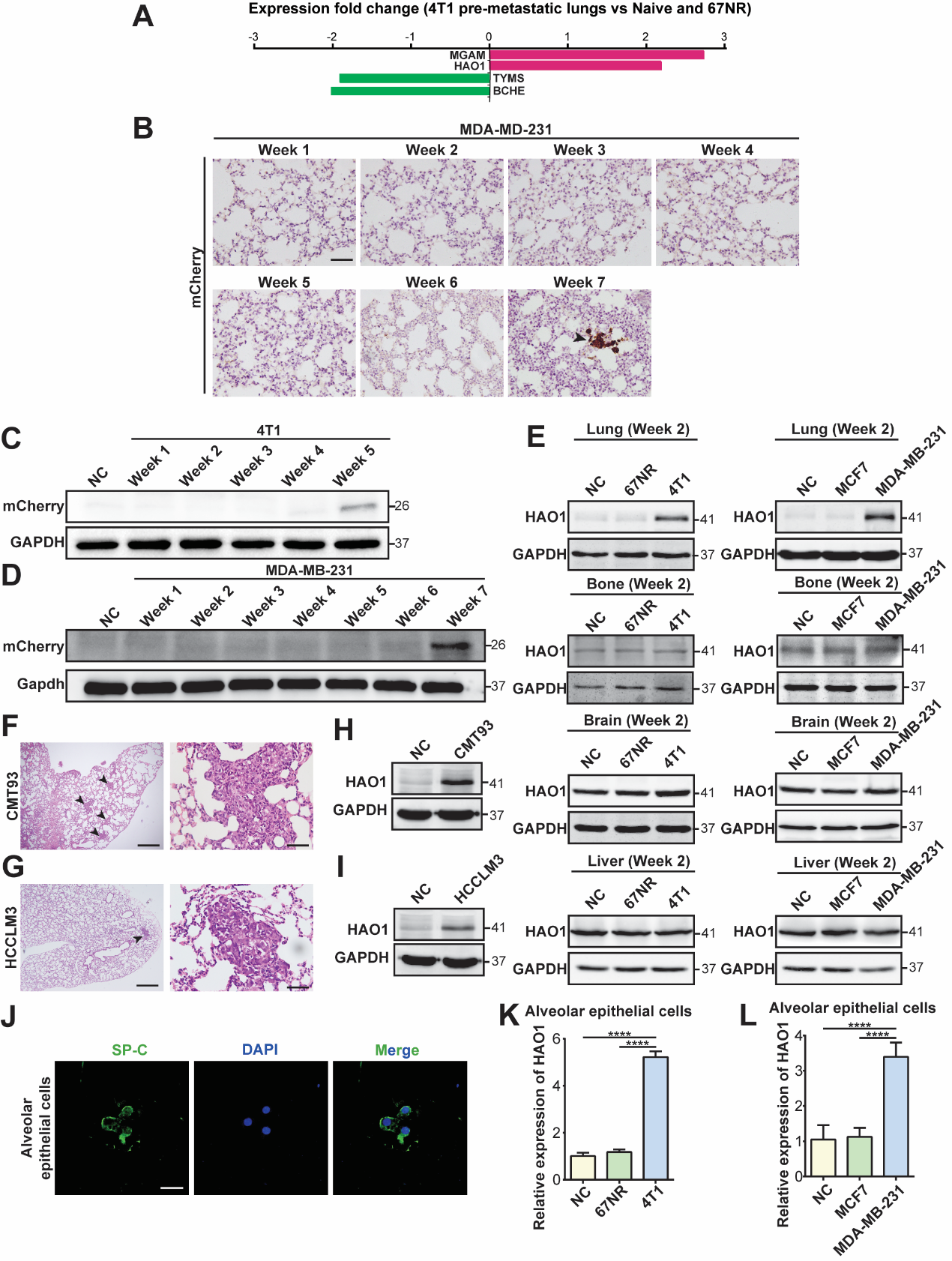


**Supplementary Figure 1.** HAO1 is up-regulated in pre-metastatic lung. **(A)** Metabolism-related genes differentially expressed in the PMN lungs of 4T1-bearing mice compared with naïve or 67NR-bearing mice (GEO accession: GSE62817). **(B)** Identification of tumor foci in lungs from mice bearing mCherry-expressing MDA-MB-231 cells for 1 to 7 weeks. The tumor foci were identified by mCherry staining of lung histologic sections. Scale bar, 20 µm. **(C)** Western blot analysis of mCherry expression in lungs from mice bearing mCherry-expressing 4T1 cells for 1 to 5 weeks. **(D)** Western blot analysis of mCherry expression in lungs from mice bearing mCherry-expressing MDA-MB-231 cells for 1 to 7 weeks. Scale bar, 20 µm. **(E)** Western blot analysis of HAO1 expression in lungs, brains, bones and livers from control mice and mice bearing 67NR, 4T1, MCF7, MDA-MB-231 cells for 2 weeks. **(F)** Image of lung metastases from mice orthotopically implanted with CMT93. Scale bar in left panels represents 200 µm. Scale bar in right panels represents 20 µm. **(G)** Image of lung metastases from mice orthotopically HCCLM3. Scale bar in left panels represents 200 µm. Scale bar in right panels represents 20 µm. **(H)** Western blotting analysis of HAO1 expression in lungs from control mice and mice bearing CMT93 cells for 2 weeks. **(I)** Western blotting analysis of HAO1 expression in lungs from control mice and mice bearing HCCLM3 cells for 2 weeks. **(J)** Immunofluorescence image of SP-C expression (green) in primary alveolar epithelial cells isolated from mice. Scale bar, 20 µm. **(K)** QPCR analysis of HAO1 expression in alveolar epithelial cells cocultured with 67NR, 4T1 cells. Means ± s.e.m are provided (n=3). **(L)** QPCR analysis of HAO1 expression in alveolar epithelial cells cocultured with MCF7 and MDA-MB-231 cells. Means ± s.e.m are provided (n=3). ****P<0.0001 according to the two-tailed Student’s t-test.


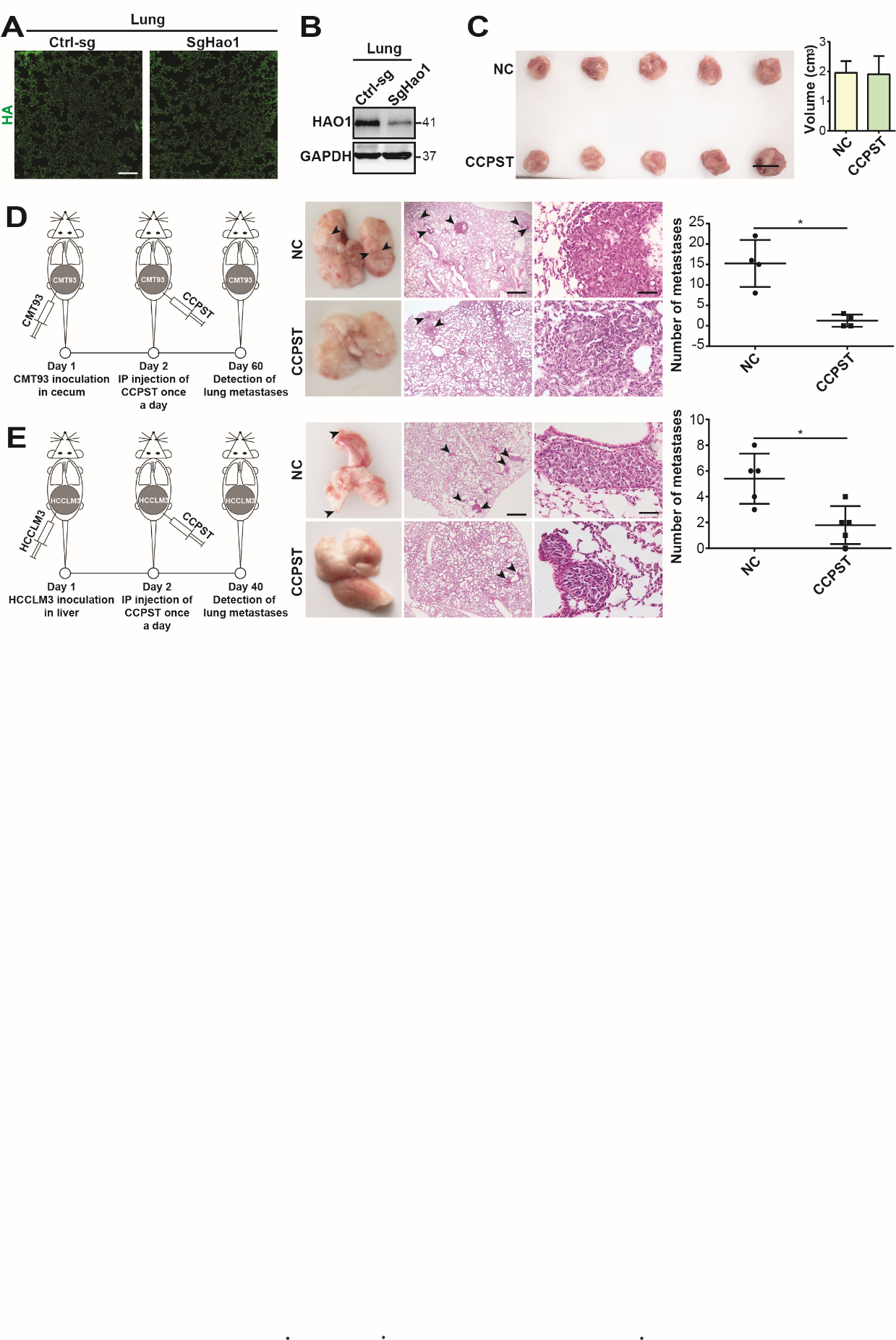


**Supplementary Figure 2.** The effect of pharmacologic inhibition or knockdown of HAO1. **(A)** The efficiency of AAV6-control sgRNA or AAV6-Hao1 sgRNA infection were determined by detecting HA expression (green) in mice lungs. Scale bar, 100 µm. **(B)** Detection of HAO1 expression in lungs from 4T1-bearing mice infected with AAV6-control sgRNA or AAV6-Hao1 sgRNA. **(C)** The volume of primary tumor from 4T1-bearing mice injected with CCPST. Scale bar, 1 cm. Means ± s.e.m are provided (n=5). **(D)** Effect of CCPST treatment on lung metastasis in mice bearing CMT93 cells. Means ± s.e.m are provided (n=4). The scale bar in the middle panels represents 200 µm. The scale bar in the right panels represents 20 µm. **(E)** Effect of CCPST treatment on lung metastasis in mice bearing HCCLM3. Means ± s.e.m are provided (n=5). The scale bar in the middle panels represents 200 µm. The scale bar in the right panels represents 20 µm. *P<0.05 according to two-tailed Student’s t-test.

**
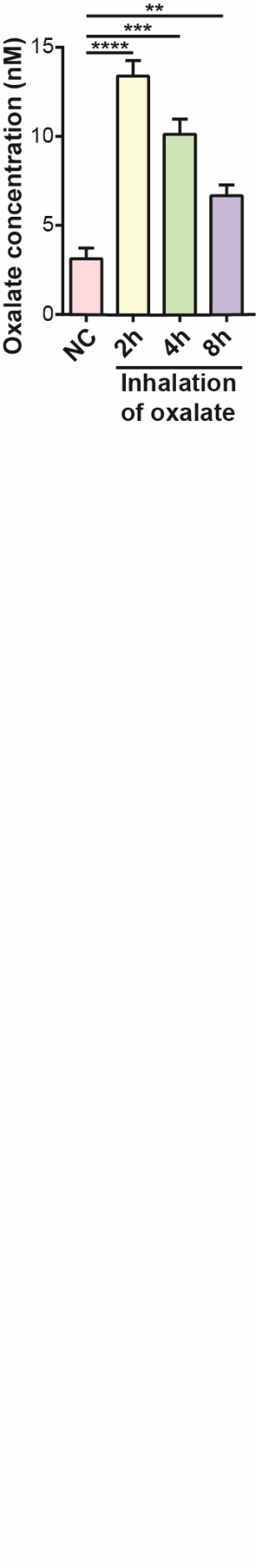
**

**Supplementary Figure 3.** Oxalate inhalation leads to oxalate accumulation in mice lungs. Detection of the oxalate concentration in mice lungs at 2, 4, 8 hours after inhalation of oxalate. Means ± s.e.m are provided (n=3). **P<0.01, ***P<0.001, ****P<0.0001 according to the two-tailed Student’s t-test.


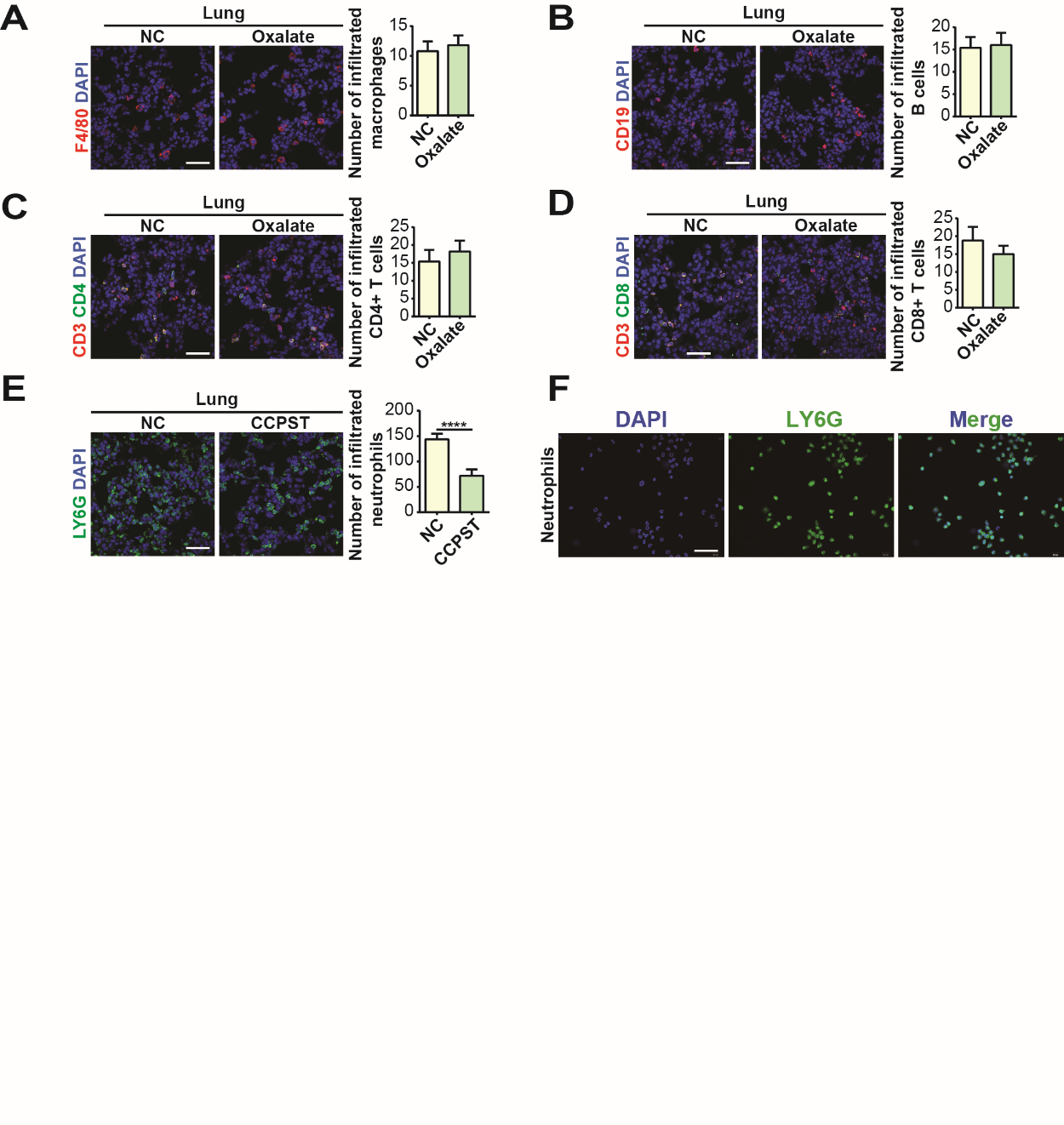


**Supplementary Figure 4.** Effect of oxalate inhalation on inflammatory cellular composition within mice lungs. **(A)** Images and quantification of macrophages (labeled by F4/80, red) in the lungs from control mice and mice received a three-daily inhalation of oxalate for one week. Scale bars, 20 µm. Means ± s.e.m are provided (n = 5). **(B)** Images and quantification of B cells (labeled by CD19, red) in the lungs from control mice and mice received a three-daily inhalation of oxalate for one week. Scale bars, 20 µm. Mean ± s.e.m are provided (n = 5). **(C)** Images and quantification of CD4+ T cells (labeled by CD4+ (green) and CD3+ (red) ) in the lungs from control mice and mice received a three-daily inhalation of oxalate for one week. Scale bars, 20 µm. Means ± s.e.m are provided (n = 5). **(D)** Images and quantification of CD8+ T cells (labeled by CD8+ (green) and CD3+ (red) ) in the lungs from control mice and mice received oxalate inhalation for one week. Scale bars, 20 µm. Mean ± s.e.m are provided (n = 5). **(E)** Images and quantification of neutrophils (labeled by LY6G, green) in the lungs from 4T1-bearing mice injected with CCPST. Scale bars, 20 µm. Means ± s.e.m are provided (n = 5). **(F)** Immunofluorescence image of LY6G expression (green) in primary neutrophils derived from mice bone marrow. Scale bar, 20 µm. ****P<.0001 according to two-tailed Student’s t-test.


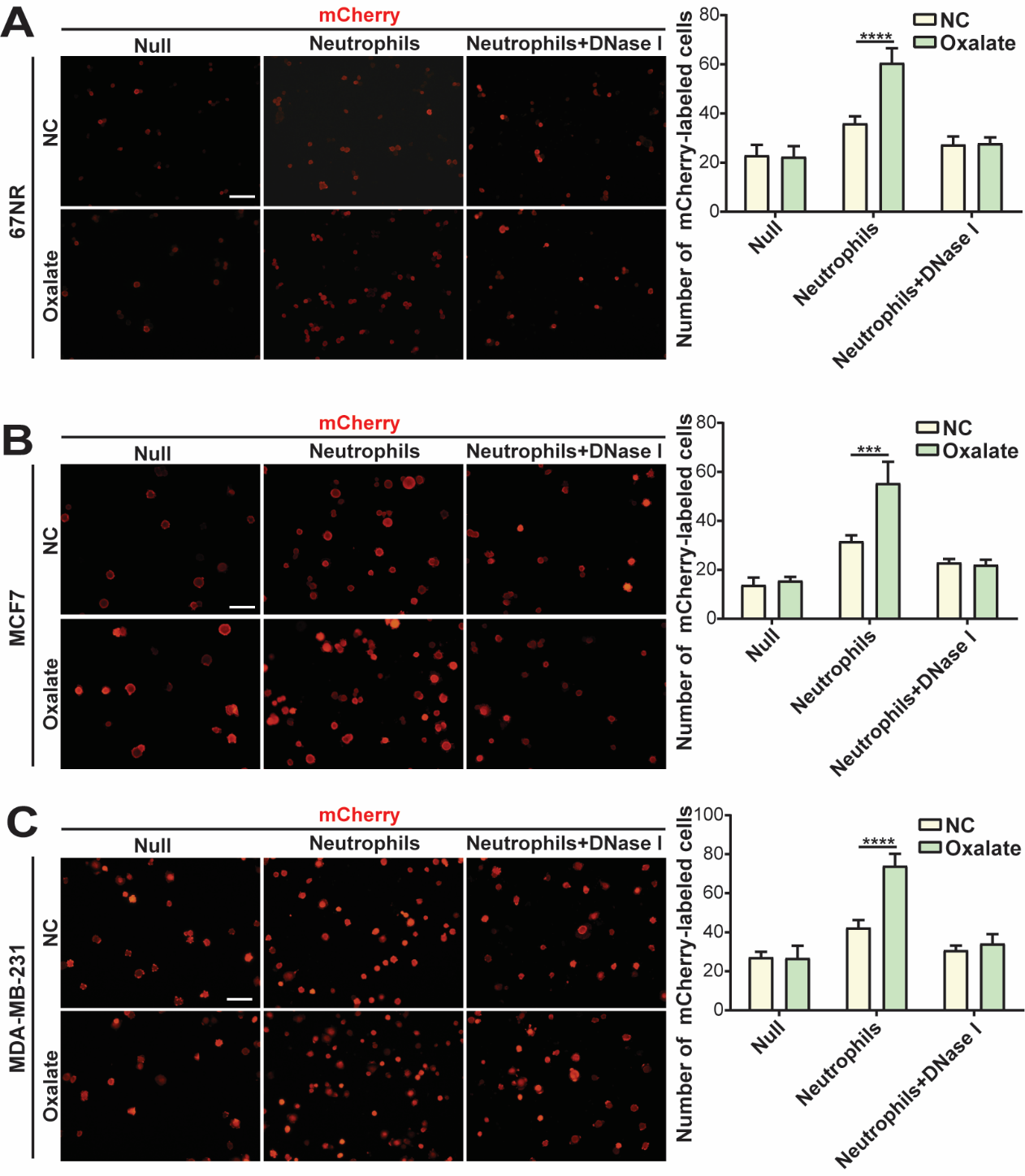


**Supplementary Figure 5.** Oxalate-induced NET formation promotes the adhesion of cancer cells. **(A)** Effect of PBS, oxalate, PBS + neutrophils, oxalate + neutrophils, PBS + neutrophils + DNase I, oxalate + neutrophils + DNase I treatment on the number of 67NR cells (labeled by mCherry, red) adhering to the bottom of the wells. Scale bars, 20 µm. Means ± s.e.m are provided (n = 5). **(B)** Effect of PBS, oxalate, PBS + neutrophils, oxalate + neutrophils, PBS + neutrophils + DNase I, oxalate + neutrophils + DNase I treatment on the number of MCF7 cells (labeled by mCherry, red) adhering to the bottom of the wells. Scale bars, 20 µm. Means ± s.e.m are provided (n = 5). **(C)** Effect of PBS, oxalate, PBS + neutrophils, oxalate + neutrophils, PBS + neutrophils + DNase I, oxalate + neutrophils + DNase I treatment on the number of MDA-MB-231 cells (labeled by mCherry, red) adhering to the bottom of the wells. Scale bars, 20 µm. Means ± s.e.m are provided (n = 5). ***P<0.001, ****P<0.0001 according to the two-tailed Student’s t-test.


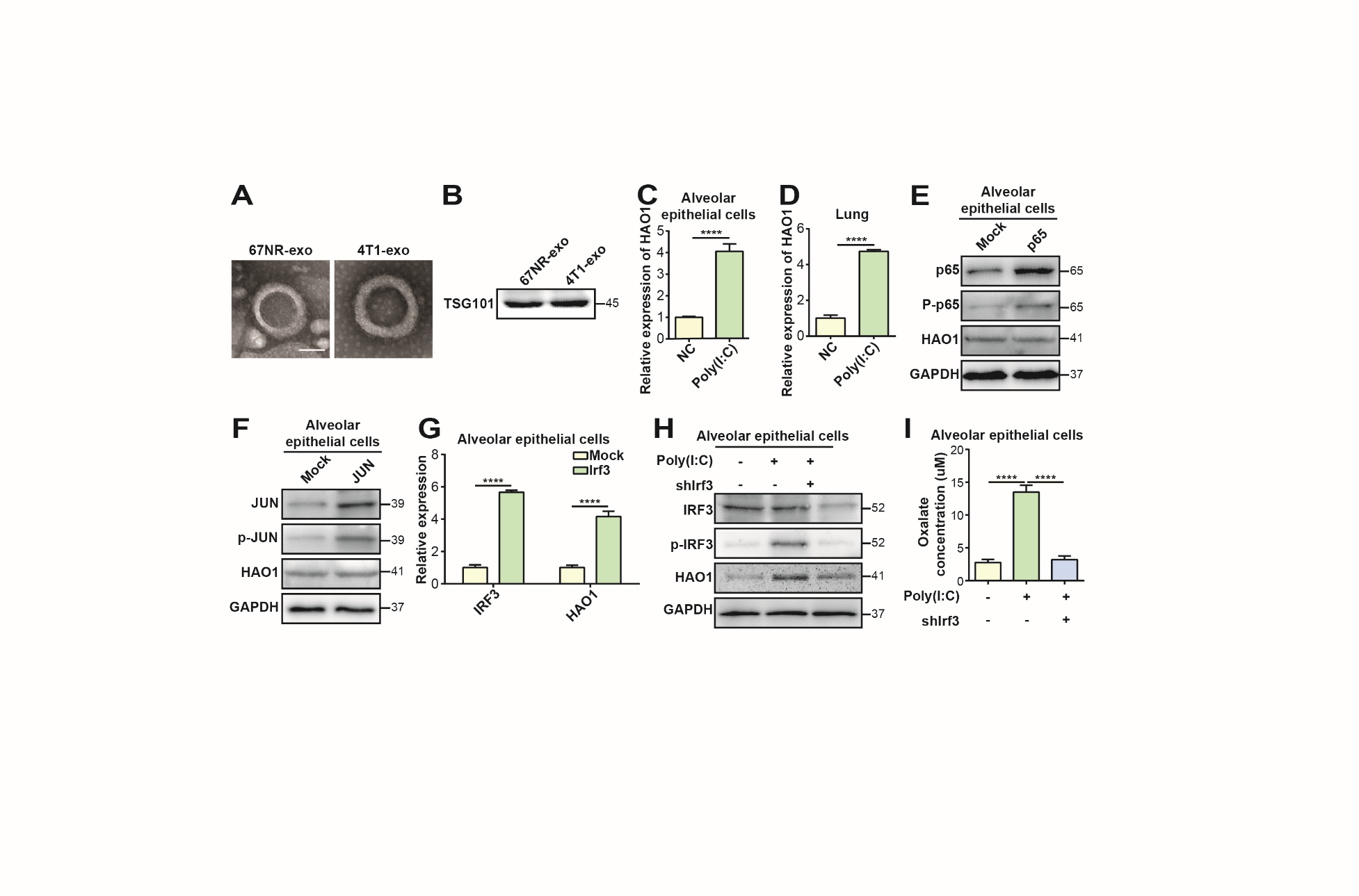


**Supplementary Figure 6.** 4T1 exosomes induced HAO1 expression and oxalate production via TLF3-IRF3 signaling. **(A)** Transmission electron microscopy of exosomes derived from 67NR and 4T1 cells. Scale bar, 50 nm. **(B)** Western blot analysis of TSG101 expression (exosomes marker) in exosomes derived from 67NR and 4T1 cells. **(C)** Effect of poly (I:C) treatment on HAO1 expression in alveolar epithelial cells by qPCR. Means ± s.e.m are provided (n = 3). **(D)** Effect of poly (I:C) inhalation on HAO1 expression in mice lung by qPCR. Means ± s.e.m are provided (n = 3). **(E)** Effect of p65 over-expression on p65, p-p65 and HAO1 expression in alveolar epithelial cells by Western blot analysis. **(F)** Effect of JUN over-expression on JUN, p-JUN and HAO1 expression in alveolar epithelial cells by Western blot analysis. **(G)** Effect of IRF3 over-expression on IRF3 and HAO1 expression in alveolar epithelial cells by qPCR. Means ± s.e.m are provided (n = 3). **(H)** Detection of HAO1 expression in alveolar epithelial cells treated with poly (I:C) or poly (I:C) + shIRF3 by qPCR. Means ± s.e.m are provided (n = 3). **(I)** Detection of oxalate production in alveolar epithelial cells treated with poly (I:C) or poly (I:C) + shiIRF3. Means ± s.e.m are provided (n = 3). ****P<.0001 according to two-tailed Student’s t-test.

**Supplementary Table 1.** Sequences of AAV6-control sgRNA or AAV6-Hao1 sgRNA.

| **Primer** | **Sequence(5’→3’)** |
| --- | --- |
| AAV6-control sgRNA | GGCTTCGCGCCGTAGTCTTA |
| AAV6-Hao1 sgRNA-1 | GTGTTTCTTACCTAGAAAATGC |
| AAV6-Hao1 sgRNA-2 | GCAGCAACGTTGCGAAGCATCC |
| AAV6-Hao1 sgRNA-3 | GAGCTGGCCCAGAGGCACTTCG |

**Supplementary Table 2.** Sequences of control sgRNA or sg-Tlr3.

| **Primer** | **Sequence(5’→3’)** |
| --- | --- |
| Control sgRNA | GCACTACCAGAGCTAACTCA |
| Sg-Tlr3 | GTTGGGCGTTGTTCAAGAGG |

**Supplementary Table 3.** Sequences of control shRNA or shIrf3.

| **Primer** | **Sequence(5’→3’)** |
| --- | --- |
| Control shRNA | TTCTCGAACGTGTCACGT |
| ShIrf3 | CGGAAAGAAGTGTTGCGGTTA |

**Supplementary Table 4.** Primer sequences for qPCR.

| **Primer** | **Sequence(5’→3’)** |
| --- | --- |
| Hao1-F | GACCGTGAGATCAGCAGACA |
| Hao1-R | GTTCCGCACGTCATCAATGC |
| Gapdh-F | AGGTCGGTGTGAACGGATTTG |
| Gapdh-R | TGTAGACCATGTAGTTGAGGTCA |
| Mgam-F | GTGCTTGAGCCTAATGCCAG |
| Mgam-R | CTTCCACTCTCCCGTTGCAT |
| Bche-F | CAGACTCAGCATACCAAGGTAAC |
| Bche-R | CTCGGACCCTTCCGGTCTT |
| Tyms-F | GGAAGGGTGTTTTGGAGGAGT |
| Tyms-R | GCTGTCCAGAAAATCTCGGGA |
| Cox2-F | TGAGCAACTATTCCAAACCAGC |
| Cox2-R | GCACGTAGTCTTCGATCACTATC |
| Il1β-F | GCAACTGTTCCTGAACTCAACT |
| Il1β-R | ATCTTTTGGGGTCCGTCAACT |
| Il6-F | TAGTCCTTCCTACCCCAATTTCC |
| Il6-R | TTGGTCCTTAGCCACTCCTTC |
| Tnfα-F | CCCTCACACTCAGATCATCTTCT |
| Tnfα-R | GCTACGACGTGGGCTACAG |
| Bv8-F | GCCCCGCTACTGCTACTTC |
| Bv8-R | CCCCGTGCAGACACTAACTTT |
| Mmp9-F | CTGGACAGCCAGACACTAAAG |
| Mmp9-R | CTCGCGGCAAGTCTTCAGAG |
| S100a8-F | AAATCACCATGCCCTCTACAAG |
| S100a8-R | CCCACTTTTATCACCATCGCAA |
| S100a9-F | ATACTCTAGGAAGGAAGGACACC |
| S100a9-R | TCCATGATGTCATTTATGAGGGC |
| Irf3-F | GAGAGCCGAACGAGGTTCAG |
| Irf3-R | CTTCCAGGTTGACACGTCCG |
